# Cardiac mechanisms for low aerobic power in anthracycline treated, older, long-term breast cancer survivors

**DOI:** 10.1186/s40959-022-00134-1

**Published:** 2022-04-11

**Authors:** Rhys I. Beaudry, Mark J. Haykowsky, James P. MacNamara, Wesley J. Tucker, Roshni Rao, Barbara Haley, Satyam Sarma

**Affiliations:** 1 University of Texas at Arlington, Arlington, TX USA; 2 University of Alberta, Edmonton, Canada; 3 Institute for Exercise and Environmental Medicine, Texas Health Presbyterian Hospital, Dallas, TX USA; 4 Department of Internal Medicine, University of Texas Southwestern Medical Center, Dallas, TX USA; 5 Texas Woman’s University, Houston, TX USA; 6 Columbia University Irving Medical Center, New York Presbyterian, New York, NY USA; 7 Department of Surgery, University of Texas Southwestern Medical Center, Dallas, TX USA

**Keywords:** Breast cancer, Anthracycline, Cardiac function, Exercise, Aerobic capacity, VO_2peak_

## Abstract

Breast cancer survivors have reduced peak aerobic capacity (VO_2peak_) which may be related to latent or lingering chemotherapy induced cardiac damage. Nine, older (67 ± 3 years), long-term survivors (9.8 years) of anthracycline based chemotherapy and age- and sex-matched healthy controls were recruited and tested to determine whether: i) VO_2peak_ remains reduced in long-term survivorship; and ii) reductions in VO_2peak_ are due to cardiac dysfunction. VO_2peak_ was significantly reduced in breast cancer survivors relative to healthy controls (15.9 ± 2.0 vs 19.9 ± 3.1 ml/kg/min, *p* = 0.006), however the heart rate and stroke volume responses to exercise were normal (heart rate reserve; 88 ± 9 vs 85 ± 10 bpm, *p* = 0.62: stroke volume reserve; 13 ± 6 vs 13 ± 9 ml,*p* = 0.94). These findings indicate low-normal ventricular size in long-term breast cancer survivors, but normal reserve function.

Breast cancer (BC) survivors have reduced peak aerobic capacity (VO_2peak_) which is associated with increased cardiovascular and all-cause mortality [1]. The “*Compounding Risk Model”* of older age, pre-existing cardiovascular risk factors, detrimental lifestyle patterns and treatment toxicity culminates in elevated cardiovascular risk [2]. A limitation of prior studies measuring VO_2peak_ is the primary focus on middle-aged BC survivors in whom testing was performed within 5 years of completion of adjuvant therapy [3]. Whether reductions in VO_2peak_ persist and possibly accelerate with aging is unknown [2]. To address this, we measured VO_2peak_ and its Fick determinants in older (≥ 60 years), female long-term (> 5 years) BC survivors previously treated with cardiotoxic anthracycline chemotherapy (*n* = 9, mean age: 67 years, mean time post-anthracycline chemotherapy completion: 9.8 years) and age-and-sex-matched healthy control subjects (*n* = 8). We hypothesized that VO_2peak_ would be reduced in older long-term BC survivors compared to control subjects and investigated whether this was related to impaired cardiac output (Qc).

Patients with breast cancer were recruited from the UT Southwestern Comprehensive Cancer Center clinic. Control subjects were recruited from a volunteer research registry. Cardiopulmonary exercise testing at two sub-maximal and maximal work rates was performed on a semi-recumbent cycle ergometer (Lode, Netherlands) during which VO_2_ (Douglas bag technique), Qc (acetylene rebreathing, AR), heart rate (Lead II ECG), and blood pressure (Tango M2, SunTech Medical, Morrisville, USA) were measured. Echocardiography (Epiq 7, Philips, Eindhoven, Netherlands) was performed at rest and 20 watts (W) to measure 3D left ventricular (LV) volumes, ejection fraction, and diastolic function. All data are presented as mean and standard deviation. Group differences were tested using Student’s t-tests with an alpha level set at *p* < 0.05 using SPSS (version 24, IBM SPSS, Armonk, USA). A priori, this study was powered to detect a 5 ± 3.5 ml/kg/min group difference in VO_2peak_ (α = 0.05, β = 0.20) This study was approved by the University of Texas Southwestern Ethics Review Board (STU112016–029).

Age, body mass index and self-reported weekly physical activity were similar between BC and control subjects (Table 1). Resting end-diastolic volume, stroke volume (SV), Qc, and sub-maximal (20 and 40 W) SV were significantly lower in BC survivors compared to controls. Peak power output, VO_2_, SV, and Qc were significantly lower in BC survivors with no difference between groups for peak heart rate, arterial-venous oxygen difference, mean arterial pressure, or the ΔQc/ΔVO_2_ slope (BC: 6.7 ± 0.7 vs. Controls: 6.3 ± 0.5, *p* = 0.20, Table 1).

**Table 1:** Subject Characteristics, Rest and Exercise Hemodynamics and Oxygen Uptake

	BC (*n* = 9)	Control (*n* = 8)	*P* Value
Age (years)	67 (3)	67 (5)	0.96
BSA (m^2^)	1.77 (0.14)	1.72 (0.07)	0.38
BMI (kg/m^2^)	27.6 (4.3)	24.3 (2.2)	0.08
Self-Reported Physical Activity (minutes/week)	125 (100)	126 (135)	0.96
Time post-anthracycline chemotherapy completion (years)	9.8 (5.2)	–	–
**Rest**			
VO_2_ (l/min)	0.19 (0.03)	0.20 (0.04)	0.34
VO_2_ (ml/kg/min)	2.7 (0.5)	3.0 (0.5)	0.16
Qc (l/min)	3.34 (0.40)	4.13 (0.52)	**0.003**
HR (bpm)	71 (9)	71 (8)	0.99
SV_AR_ (ml)	48 (7)	58 (8)	**0.01**
MAP (mmHg)	101 (10)	93 (13)	0.2
EDV_Echo_ (ml)	64 (9)	79 (11)	**0.04**
ESV_Echo_ (ml)	30 (5)	32 (7)	0.70
EF (%)	52 (7)	60 (5)	0.06
E/e’	8.7 (2.4)	8.2 (2.1)	0.69
e’_average_	7.0 (1.7)	7.4 (2.1)	0.71
E/A	0.89 (0.09)	0.96 (0.23)	0.47
**Submaximal Exercise, 20 W**			
VO_2_ (l/min)	0.55 (0.08)	0.57 (0.07)	0.60
VO_2_ (ml/kg/min)	7.8 (1.4)	8.9 (1.2)	0.10
Qc (l/min)	6.36 (0.97)	7.24 (0.81)	0.06
HR (bpm)	94 (14)	92 (6)	0.71
SV_AR_ (ml)	68 (10)	79 (8)	**0.03**
MAP (mmHg)	110 (13)	105 (14)	0.49
EDV_Echo_ (ml)	69 (9)	91 (23)	0.07
ESV_Echo_ (ml)	28 (5)	36 (10)	0.14
EF (%)	60 (6)	61 (2)	9.72
E/e’	8.0 (1.3)	8.2 (1.8)	0.76
e’_average_	11.1 (1.7)	9.9 (1.7)	0.18
E/A	1.00 (0.22)	0.96 (0.19)	0.73
**Submaximal Exercise, 40 W**			
VO_2_ (l/min)	0.73 (0.13)	0.77 (0.09)	0.50
VO_2_ (ml/kg/min)	10.5 (2.5)	11.5 (1.6)	0.35
Qc (l/min)	7.44 (0.81)	8.01 (0.72)	0.17
HR (bpm)	112 (19)	103 (10)	0.25
SV_AR_ (ml)	68 (11)	78 (6)	**0.04**
MAP (mmHg)	116 (15)	107 (11)	0.22
**Peak Exercise**			
Power output (Watts)	81 (12)	99 (13)	**0.01**
VO_2_ (l/min)	1.11 (0.09)	1.32 (0.15)	**0.003**
VO_2_ (ml/kg/min)	15.9 (2.0)	19.9 (3.1)	**0.006**
RER	1.15 (0.07)	1.17 (0.08)	0.67
Qc (l/min)	9.49 (1.05)	11.40 (1.03)	**0.003**
a-vO_2Diff_ (ml/dL)	11.8 (0.9)	11.8 (0.8)	0.87
HR (bpm)	159 (16)	156 (7)	0.71
SV_AR_ (ml)	60 (9)	72 (9)	**0.02**
MAP (mmHg)	125 (21)	120 (11)	0.63
**Reserve**			
Qc (l/min)	6.14 (0.81)	7.21 (0.75)	**0.02**
HR (bpm)	88 (9)	85 (10)	0.62
SV_AR_ (ml)	13 (6)	13 (9)	0.94

*BSA* body surface area, *BMI* body mass index, *VO*_2_ oxygen uptake, *RER* respiratory exchange ratio, *Qc* cardiac output, *HR* heart rate, *SV* stroke volume, *AR* acetylene rebreathe, *MAP* mean arterial pressure, *Echo* Echocardiography, *EDV* end diastolic volume, *ESV* end systolic volume, *EF* ejection fraction, *E/e’* ratio of early diastolic mitral inflow to myocardial tissue velocity, *E/A* ratio of early diastolic to late mitral inflow, *a-vO*_2_ *Diff* arterial-venous oxygen content difference

The novel findings of this study are: i) a lower VO_2peak_ in older, long-term BC survivors, ii) a lower rest SV and peak exercise SV and Qc, and iii) similar relative matching of Qc for achieved metabolic work (ΔQc/ΔVO_2_ slope) between older long-term BC survivors and controls.

BC survivors had severe and marked exercise intolerance as demonstrated by 20% lower VO_2peak_ compared to age-and-sex-matched controls. Their mean VO_2peak_ was similar to the threshold level required for full and independent living (e.g. 15.4 ml/kg/min), and occurred a decade earlier than expected for healthy sedentary women without a history of BC (e.g. 77 years of age) [4]. This accelerate physiological aging was accompanied by reduced cardiac size (EDV, ESV, SV) despite indicators of LV filling pressure and relaxation (E/e’, E/A ratio) being normal. It is unknown whether the smaller SV and EDV with normal diastolic pressure gradients at rest and exercise is a consequence of smaller geometric chamber properties or cardiac atrophy and altered tissue characteristics (fibrosis and stiffening) consistent with mechanisms of anthracycline cardiotoxicity.

In contrast to the marked impairment in VO_2peak_ in BC, the cardiac responses to submaximal and peak exercise appeared normal. While SV was lower in BC at rest, SV reserve (rest to peak exercise) was not different between groups, nor was the ΔQc/ΔVO_2_ slope, indicating appropriate regulation of cardiac output for the metabolic demands of exercise. Rather, a close link persists between gross ventricular size and peak exercise capacity whereby a “small heart” phenotype is observed in our BC survivors accounting for reduced peak Qc. In our sample, the mean LV EDV for BC survivors fell towards the lower end of normal, while controls averaged near the upper end of normal. We have reported similar findings of low cardiac volumes and output relative to body size in BC patients *prior* to receiving cardiotoxic anthracycline therapy- the obvious scapegoat for cardiac atrophy and impaired exercise capacity [5]. It remains possible that a healthy bias effect may exist within both study arms; particularly as BC participants were ~ 10 years beyond anthracycline therapy, they are by definition healthier than the subset of BC patients who do not survive a decade beyond diagnosis and treatment. Further work is needed to understand the relationship between anthracycline exposure and aging and whether these are synergistic in accelerating cardiac atrophy and declines in cardiorespiratory fitness.

## Data Availability

The dataset used during the current study are available from the corresponding author on reasonable request.

## Abbreviations

BC: Breast Cancer
VO_2peak_: Peak oxygen uptake
Qc: Cardiac output
AR: Acetylene Rebreathe
W: Watts
LV: Left Ventricle
SV: Stroke Volume
ED/ES V: End-Diastolic/End-Systolic Volume
